# Thumb-loops up for catalysis: a structure/function investigation of a functional loop movement in a GH11 xylanase

**DOI:** 10.5936/csbj.201207001

**Published:** 2012-07-01

**Authors:** Gabriel Paës, Juan Cortés, Thierry Siméon, Michael J. O'Donohue, Vinh Tran

**Affiliations:** a CNRS, FRE3478 UFIP, Faculté des Sciences et des Techniques, 2 rue de la Houssinière, F-44322 Nantes, France; b University of Nantes, FRE3478 UFIP, Faculté des Sciences et des Techniques, 2 rue de la Houssinière, F-44322 Nantes, France; c INRA, UMR614 FARE, 2 esplanade Roland Garros, F-51686 Reims, France; d University of Reims Champagne-Ardenne, UMR614 FARE, 2 esplanade Roland Garros, F-51686 Reims, France; e CNRS, LAAS, 7 avenue du colonel Roche, F-31400 Toulouse, France; f University of Toulouse, LAAS, F-31400 Toulouse, France; g INRA, UMR792 LISBP, 137 avenue de Rangueil, F-31077 Toulouse, France; h INSA, UMR792 LISBP, 137 avenue de Rangueil, F-31077 Toulouse, France

**Keywords:** Structure-function relationship, xylanase thumb-loop, molecular modeling, path planning algorithms

## Abstract

Dynamics is a key feature of enzyme catalysis. Unfortunately, current experimental and computational techniques do not yet provide a comprehensive understanding and description of functional macromolecular motions. In this work, we have extended a novel computational technique, which combines molecular modeling methods and robotics algorithms, to investigate functional motions of protein loops. This new approach has been applied to study the functional importance of the so-called thumb-loop in the glycoside hydrolase family 11 xylanase from *Thermobacillus xylanilyticus* (Tx-xyl). The results obtained provide new insight into the role of the loop in the glycosylation/deglycosylation catalytic cycle, and underline the key importance of the nature of the residue located at the tip of the thumb-loop. The effect of mutations predicted *in silico* has been validated by *in vitro* site-directed mutagenesis experiments. Overall, we propose a comprehensive model of Tx-xyl catalysis in terms of substrate and product dynamics by identifying the action of the thumb-loop motion during catalysis.

## Introduction

Dynamics is an intrinsic feature of enzymes that promotes catalysis in different ways [1]. Enzyme motions can cause the exclusion of water molecules from the active site, optimally position catalytic groups, provide substrate selectivity or even prevent catalytic intermediates from escaping [2]. Enzyme dynamics was first evidenced using X-ray crystallography to examine enzyme structures in the presence or absence of substrates, inhibitors, etc. This revealed that some enzymes adopt a different conformation when bound to a ligand. Other analytical techniques such as neutron and small angle scattering [3, 4], NMR spectroscopy [5], and hydrogen-deuterium exchange [6] have since refined our understanding of enzyme dynamics. Moreover, enzyme kinetics and mutational studies have provided complementary data that confirm the importance of enzyme motions for activity [7–9]. Despite significant progress of computational methods over the last decades, the computer-assisted prediction and analysis of protein motions is still challenging.

Molecular dynamics (MD) simulations [10] require massive calculation times, particularly for large atomic assemblies such as protein/substrate complexes. Furthermore, the time-scale of MD simulations is ill-adapted to the biological processes under study, which occur on timescales ranging from nanoseconds to seconds. Therefore, to explore the main features of intrinsic protein motions responsible for activity, a new computational approach [11] that employs robotics algorithms was recently introduced as an alternative to MD simulations. The key principle behind this methodology is the separation of the exploration of protein conformations into two distinct stages. The first stage consists of a geometric filtering operation that is driven by a path planning algorithm, originating from robotics. This first stage explores feasible motions of an articulated hard-sphere model of the molecular system. In a second stage, geometrically feasible transition pathways obtained from the first stage are submitted to classical molecular modeling techniques, which are used to refine the motions considering a molecular force field. The strength of this two-stage approach lies in the fact that large amplitude motions can be studied using quite modest computational resources [11–13]. Conversely, results are assumed to be less accurate than rigorous MD simulations, and should be analyzed mainly qualitatively. Nevertheless, the rapidity and efficacy of geometric exploration also enables the study of various conformational pathways in order to perform statistical analyses of results, which can be more meaningful than the analysis of a single MD trajectory. This two-stage approach has been successfully applied to the study of protein loop motions and ligand-protein access pathways [11, 13–15]. Related methods, based on a similar two-step process, have also been used for computing other types of conformational transitions in proteins [16, 17], and for modeling protein conformational ensembles [18, 19]. In the present study, we have adopted such a two-stage approach to investigate the dynamics of a prominent loop in the GH11 xylanase from *Thermobacillus xylanilyticus* (Tx-xyl) [20, 21].

Xylanases hydrolyze β-(1,4) linkages between D − xylosyl moieties, mainly encountered in xylans, which are the major hemicellulose components of many plant cell walls. In particular, xylans are abundant components of agricultural co-products such as brans, straws, cobs and bagasse [22, 23] and constitute a major polysaccharide component of hardwoods [24]. In addition to their current industrial uses (e.g. food and drinks sector, animal feed processing and paper pulping), xylanases will be useful for biorefining [25–27]. However, whatever the targeted application, the improvement of the catalytic properties of xylanases is an overarching aim for research in this area.

Regarding Tx-xyl, the action of this enzyme on lignocellulosic biomass such as wheat straw and bran has already been studied [28–32] and engineering of this enzyme has led to improvements in hydrolysis yields [33]. However, a better understanding of the subtle features of Tx-xyl-mediated catalysis is required to pursue a rational engineering strategy.

**Figure 1:**
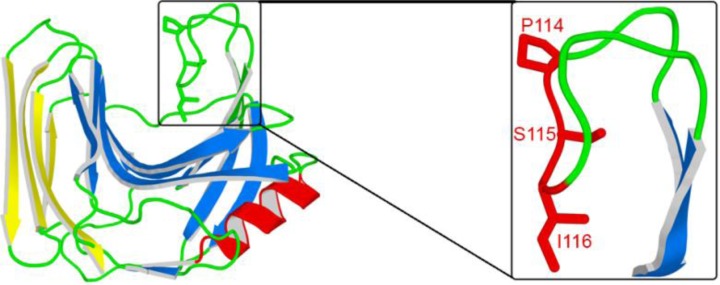
**Structure of Tx-xyl (left) and of the thumb-loop (right).** All molecular structures were drawn with PyMOL 0.98 (Delano, 2004).

All xylanases in the CAZy [34] GH11 family display a catalytic domain that has jelly-roll architecture and a characteristic long loop between the β-strands B7 and B8, which forms a striking structural feature. The overall structure of the catalytic domain has been likened to a partially folded right hand, with the long loop representing the thumb. This β7-β8 loop, which is positioned above the active site of GH11 xylanases (Figure 1), has been extensively studied, notably with regard to its interactions with proteinaceous inhibitors [35, 36] and because of its supposed mobility.

The first actual evidence of thumb-loop mobility was provided by the structural analysis of the GH11 xylanase XYNII from *Hypocrea jecorina* in the presence of epoxy-alkyl-xylosides. Different thumb-loop conformations were associated with the bound and unbound forms [37]. However, a similar study performed on the GH11 xylanase from *Bacillus circulans* failed to confirm these findings [38, 39]. Nevertheless, MD simulations of XYNII [40] evidenced three consecutive thumb-loop conformations, consistent with the completion of a catalytic cycle. The first putative conformation (B) would supposedly facilitate ligand binding to the active site, the second closed-conformation (C) would block the bound ligand in the active site and the third conformation (L) would allow product release. Although simple, this postulate is consistent with experimental evidence that has shown that the thumb-loop is a key element of substrate selectivity in Tx-xyl [41] and with recent crystallographic data for a mutant (D11F) of the GH11 xylanase from *Bacillus subtilis* that has revealed the structure of the thumb-loop in an open conformation [42]. Previously, molecular dynamics have also described a temperature-dependent thumb-loop motion [43, 44], but it is difficult to determine to what extent these results can be used to understand the role of the thumb in catalysis, since these used an unrealistically small substrate (xylobiose) and primarily investigated the role of temperature on possible conformational changes in the enzyme on a nanosecond time-scale, with no correlation to *in vitro* data.

In this study, to acquire new insight into the role of the thumb-loop in the catalytic mechanism of GH11 xylanases and thus to go beyond the scope of previous MD studies, we have applied the aforementioned approach, involving robotics algorithms and classical molecular modeling techniques. More precisely, the method has been used to simulate (open-close) movements of the thumb-loop in the wild-type Tx-xyl and in some variants, and to study how such movements may affect a realistically-sized substrate occupying the full catalytic cleft. The results indicate that the thumb-loop is important for product release and underline the fact that this function is sensitive to amino acid alterations at the tip of the thumb-loop. In a novel way, we have confronted the results obtained from *in silico* analyses with experimental data derived from the *in vitro* site-directed mutagenesis of the thumb-loop of Tx-xyl. Overall, this study allows us to propose a model for Tx-xyl-mediated catalysis, which accounts for the dynamics of the thumb-loop motion.

## Experimental Procedures

### Overview of the computational method

**Figure 2:**
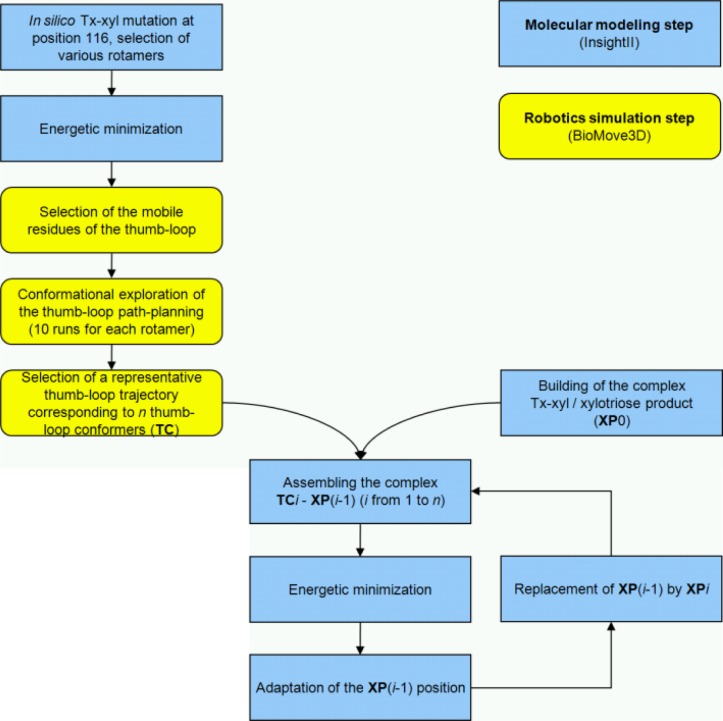
**Flow chart describing the general procedure steps applied to explore the thumb-loop trajectories of Tx-xyl and its mutants and the subsequent motion of the xylotriose reaction product.** Molecular modeling steps and robotics simulation steps are distinguished.

An extension of the robotics-based method for computing protein loop motions [11, 12] was developed, which computes coupled loop-ligand (substrate/product) motions. The procedure consisted of two main stages. In the first stage, loop motions were computed using a mechanistic model of the protein (without the ligand) and path-planning algorithms. In the second stage, the ligand was added to the model and its conformation was iteratively adapted for each frame of the pre-computed loop motion by energy minimization. The various steps of the procedure, summarized in Figure 2, are described below. In the present work, this new computational method was applied to the model of native Tx-xyl and several mutants in order to investigate the role of the thumb-loop in the expulsion of xylotriose product.

### Tx-xyl structure and in silico mutations

Although absent from the Protein Data Bank [45], the crystal structure of Tx-xyl (Figure 1) has already been solved [46]. From this structure, models of Tx-xyl variants were created by replacing the residue at the tip of the thumb-loop (Ile116) using the Biopolymer module included in Insight II (Accelrys, San Diego, CA, USA). Five different residues (Ala, Cys, Gly, Ser and Thr) were incorporated at position 116. The resultant mutants were named Tx-xyl-A, Tx-xyl-C, Tx-xyl-G, Tx-xyl-S and Tx-xyl-T respectively. For each mutation, all possible starting geometries were considered depending on the number of side-chain rotamers available in the Biopolymer library. In this way, it was possible to detect mutants that did not display, in the ground-state, insurmountable steric conflicts with nearby residues and thus select the rotamers after a 1000-iteration minimization step, using the CFF91 force field in order to relax the system. Thus, for some mutations, several rotamers were kept for further calculations.

### Thumb-loop conformational exploration

Loop motions were computed using the BioMove3D prototype software [11]. This software explores the conformational space from a given initial state and reveals attainable conformational regions and the pathways that connect them. BioMove3D applies path-planning algorithms based on random search techniques and efficient geometric functions. Computed motions satisfy loop-closure constraints and steric clash avoidance between non-bonded atoms. If a set of stable hydrogen bonds is defined, a set of geometric constraints maintains these bonds during the conformational exploration.

The Tx-xyl thumb-loop (Figure 1) was defined from residue Tyr109 to residue Thr123. Two hydrogen bonds were included: Ala113 (N)···Gly120 (O) and Ser115 (O)···Gly118 (N), based on interatomic distances and bond angles between the donor, the acceptor and the hydrogen. Only the backbone and side-chains of these 15 residues were allowed to move. BioMove3D was run several times for both the native Tx-xyl and its variants altered at position 116. 10 runs were performed for each of the rotameric conformers (as defined by InsightII options) of the amino acids under study. Each continuous conformational exploration yielded a search tree describing a set of feasible motions. The conformation positioned furthest from the initial one in the direction towards the catalytic cleft was selected as final conformation, and the path (*i.e*. continuous sequence of conformations) connecting the initial and final conformations was extracted from the tree.

### Docking model of Tx-xyl in complex with xylohexaose

Starting with the structure of a GH11 from *Bacillus circulans* complexed to xylobiose [38] (PDB ID: 1BCX), a model of Tx-xyl bound to xylohexaose was generated using Insight II (Biopolymer and Discover modules) and the CFF91 force field for all calculations. To construct the xylohexaose, xylosyl moieties were added sequentially at each extremity of the complexed xylobiose, until the catalytic cleft of Tx-xyl was fully occupied from subsite (− 3) to subsite (+ 3). After each addition of a xylosyl moiety, a 10,000-iteration minimization step was performed on the atoms of the unrestrained substrate. Whenever possible, the conformations of the hydroxyl groups of the xylosyl moieties were manually modified in order to favor hydrogen bond interactions. The subsite docking energies derived from this complex model were composed of both electrostatic and van der Waals energy terms (from the unbound state) that describe interactions between xylosyl moieties and all of the surrounding amino acid sidechains, which display heavy atoms (C, O, N or S) and which lie within a distance of 4 Å. Specific interactions such as stacking or hydrogen bonding were evaluated by isolating the atoms concerned and measuring the interaction energy between them. The product of Tx-xyl catalysis was obtained by retaining only those xylosyl moieties (a xylotriose) that occupy the non-reducing subsites (– 3) to (– 1) of the enzyme cleft.

### Motions of the xylotriose product induced by loop trajectories

The computed thumb-loop trajectories (calculated for apo-enzymes) were discretized at regular steps, in such a way that the displacement of the Cα of residue 116 between consecutive frames was approximately 0.5 Å. For a given trajectory composed of *n* thumb-loop conformers, the following process was used to iteratively “adapt” the conformation of the xylotriose product to the changing thumb-loop conformation. First, the non-reducing xylotriose reaction product (derived from the docking model of the enzyme-product complex) was added to the enzyme model with the thumb-loop displaying the first conformation of the trajectory, named conformation TC1. Designating XP0 the xylotriose product at its initial (docked) conformation, the complex TC1-XP0 was submitted to constrained energy minimization (2000 iterations). The constraints concern enzyme atomic positions at a given point to the trajectory. Clearly, it does not correspond to a full induced fit between the enzyme and the substrate because of the primacy of the enzyme loop motion trajectory. This minimization procedure caused the conformation of the xylotriose product to evolve from XP0 to XP1, thus adapting the position of the product in the cleft. This operation was repeated for the *n* frames of the thumb-loop trajectory, producing at each step a complex TC*i*+1-XP*i*. At the end of the process, either the xylotriose product was expulsed by the upward motion of the thumb-loop, or it remained undisturbed in the catalytic cleft. An energetic analysis was also performed to measure the energy of the product along the trajectory, in order to discard any improbable conformation of the complex. Note that, with this protocol, the energies could be higher than those measured during classical MD simulations. Nevertheless, intermediate conformations of the enzyme-product complex are statistically probable.

### In vitro Tx-xyl mutagenesis, expression and characterization

The Tx-xyl CDS (GenBank reference: AM 237841.1) from *Thermobacillus xylanilyticus* cloned in the vector pRSETB was mutated using the oligonucleotide primers listed in Table 1 using QuickChange Site Directed Mutagenesis Kit (Stratagene, The Netherlands) [33]. The correct introduction of the mutation was confirmed by DNA sequence analysis (MWG Biotech, Germany).

**Table 1:** Oligonucleotide primers pairs used for site-directed mutagenesis. Mutated codons are in bold.

Mutations	Primer sequence (5’ → 3’)
Ile116 → Ala	GCTACAACGCACCGTCCGCTGACGGCACGCAGACG
	CGTCTGCGTGCCGTCAGCGGACGGTGCGTTGTAGC
Ile116 → Cys	GCTACAACGCACCGTCCTGCGACGGCACGCAGACG
	CGTCTGCGTGCCGTCGCAGGACGGTGCGTTGTAGC
Ile116 → Gly	GCTACAACGCACCGTCCGGAGACGGCACGCAGACG
	CGTCTGCGTGCCGTCTCCGGACGGTGCGTTGTAGC
Ile116 → Ser	GCTACAACGCACCGTCCTCCGACGGCACGCAGACG
	CGTCTGCGTGCCGTCGGAGGACGGTGCGTTGTAGC
Ile116 → Thr	GCTACAACGCACCGTCCACGGACGGCACGCAGACG
	CGTCTGCGTGCCGTCCGTGGACGGTGCGTTGTAGC

Wild type Tx-xyl and mutants thereof (Tx-xyl-A, Tx-xyl-C, Tx-xyl-G, Tx-xyl-S and Tx-xyl-T) were expressed in *Escherichia coli* and purified using established methods [33]. Enzyme purity was checked using SDS-PAGE analysis. Catalytic parameters were determined by the discontinuous monitoring of the release of reducing sugars from birchwood xylan (Sigma-Aldrich SARL, Saint-Quentin Falavier, France). A single batch of xylan was used for all experiments and the reducing sugar content in the reaction medium was measured using a colorimetric method [33, 47]. Owing to the heterogenous nature of the polymeric substrate, only apparent values of *K*_M_ were determined. Sigma Plot 6.1 (SPSS, USA) was used to compute the catalytic rate constant *k*_cat_ and values of *K*_M__(app)_.

## Results

### Docking model of the complex between a xylohexaose and Tx-xyl active site

**Figure 3:**
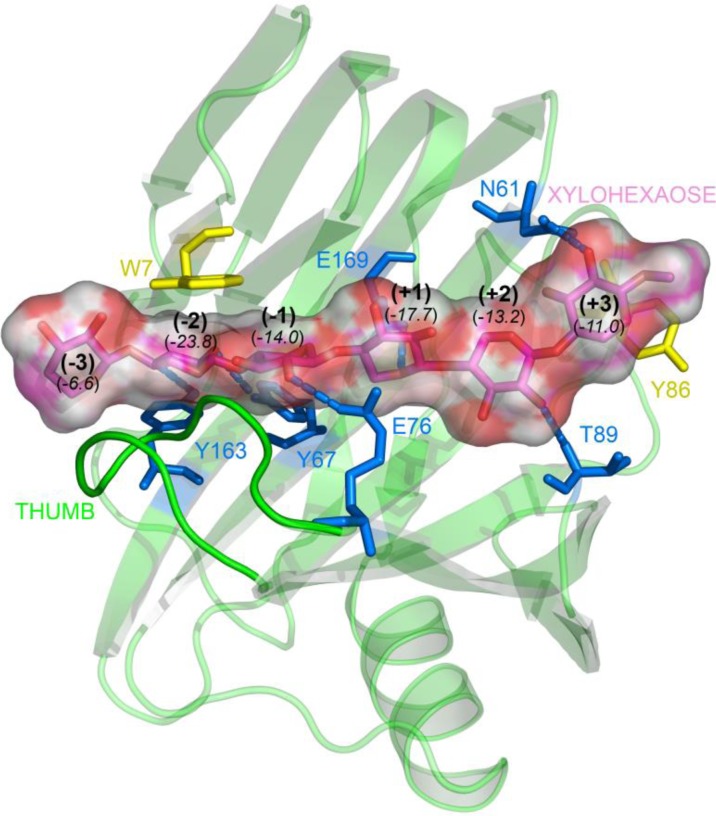
**Docking model of Tx-xyl and a xylohexaose molecule [41].** Subsite binding energies (in kcal.mol^-1^) are indicated in parentheses in italic below each subsite. Stacking residues are in yellow, hydrogen bonded residues are in blue. Substrate in glycon subsite (– 2) is perfectly accommodated between the thumb-loop and residue Trp7.

**Figure 4:**
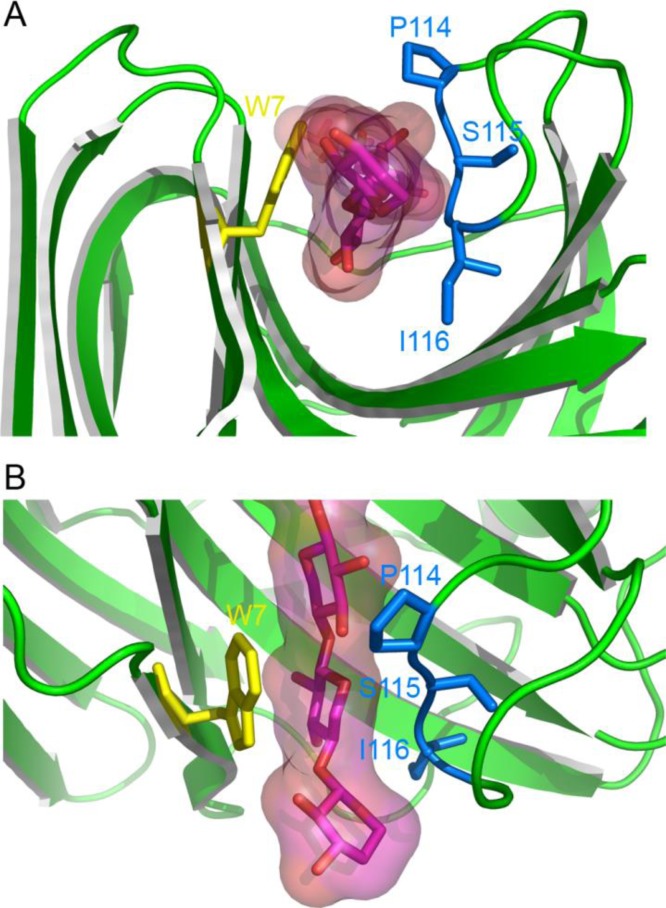
**Close-up view of the Tx-xyl docking model at the thumb-loop level.** The xylose moiety in subsite (– 2) is locked between residues Pro114 and Ile116. Views (A) along the catalytic cleft and (B) above the catalytic cleft.

Previous studies have shown that the thumb-loop contributes to subsites (− 1), (− 2) and, in one case, (– 3) [48], and reduces the overall width of the active site cleft [37–39, 49]. In particular, the xylosyl moiety bound in subsite (− 2) is held in position by sterical constraints and electrostatic interactions that are provided by the thumb-loop on one hand and by a stacking interaction with Trp7 (Tx-xyl numbering is used throughout the text) on the other. The docking of xylohexaose into the active site of Tx-xyl [41] (Figure 3) confirmed this observation and revealed that the (− 2) subsite is characterized by a high interaction energy (– 24 kcal/mol), with the Trp7 stacking interaction alone accounting for - 4.5 kcal/mol. The model also revealed that Pro114 and Ile116, both located at the tip of the thumb-loop, perfectly accommodate the substrate. Overall, these results highlight the importance of the thumb-loop for substrate binding (Figure 4).

### Thumb-loop trajectories in the apo-Tx-xyl

**Figure 5:**
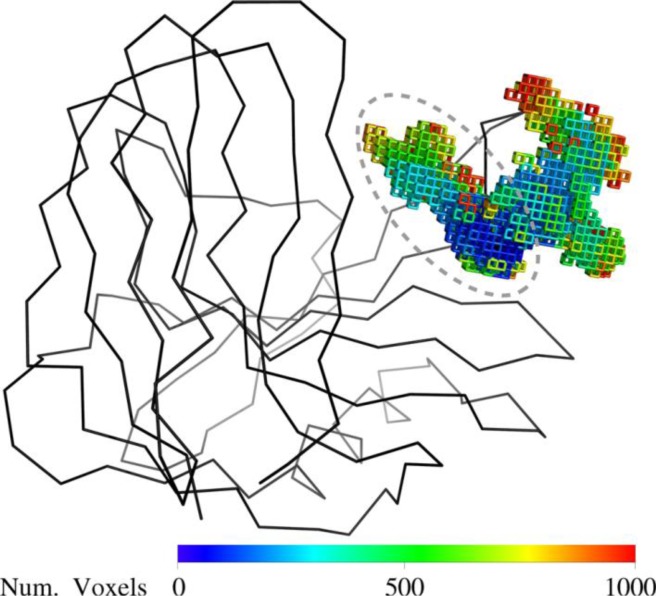
**Voxel map representing geometrically feasible motions of the Tx-xyl thumb-loop.** Voxels display the positions reached by the Ile116 Cα atom during the continuous exploration starting from the crystallographic structure. Voxels colors have been assigned depending on the chronological order of creation.

The robotics-based computational approach [11] was used to compute the possible motions of the Tx-xyl thumb-loop in the absence of substrate. Using the crystallographic structure to define the initial position of the thumb-loop, path-planning exploration revealed two main motional directions, which will be subsequently referred to as “upward” and “backward”. These motional directions are represented in Figure 5 using a voxel-map representation [50] that displays the positions reached by Ile116 Cα. The backward motion leads to a widening of the catalytic cleft, which presumably facilitates access for the substrate. Regarding the upward motion (marked with a dashed line in Figure 5), this appears to allow the thumb-loop to avoid a clash with the bulky Trp7 residue, before sliding along the length of the catalytic cleft. Intuitively, the upward motion could play an important role in product release. Therefore this motion was targeted for further analysis.

**Figure 6:**
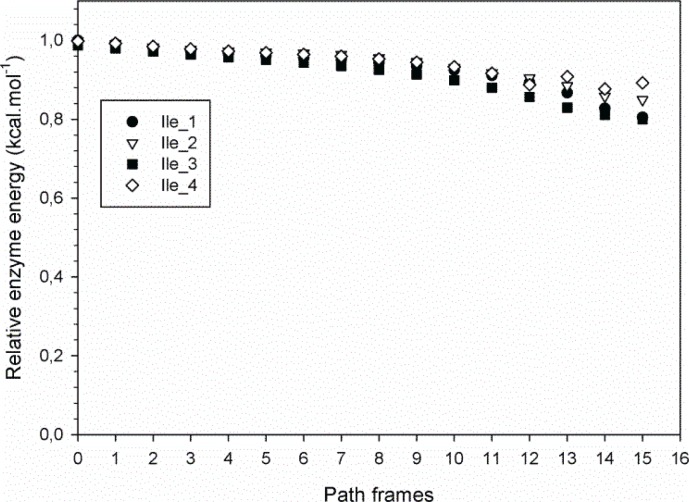
Evolution of Tx-xyl enzyme normalized energy along the upward trajectory for four different representative Ile116 rotamers.

Four trajectory sets (10 trajectories per set) were computed for the upward motion of the thumb-loop, with each set corresponding to one of the four distinct rotamers of Ile116. The 40 calculated trajectories were highly similar, suggesting that they are quite representative of the conformational fit of the thumb-loop in the catalytic cleft. Figure 6 shows the energy profile of one representative trajectory for each of the four Ile116 rotamers. Since absolute enzyme energies calculated are not realistic in comparison to classical molecular modeling calculations, they have been normalized to facilitate comparison. The very similar energy values associated with these trajectories indicate that a unique class of thumb-loop motions is generated, irrespective of the rotamer used. Moreover, because Ile116 did not undergo any major conformational changes during the whole thumb-loop motion and no drastic sterical conflicts were revealed, it can be assumed that the four rotamers are equiprobable and, thus, can be accounted for in the statistical analysis of results.

### Thumb-driven release of the xylotriose reaction product in Tx-xyl

The docking model of Tx-xyl (Figure 3) reveals that the thumb-loop only interacts with xylosyl moieties present in the glycone subsites. Accordingly, to investigate the impact of the upward motion of the thumb-loop on product release, a docking model of Tx-xyl with xylotriose bound subsites (− 3), (− 2) and (− 1) was considered.

**Figure 7:**
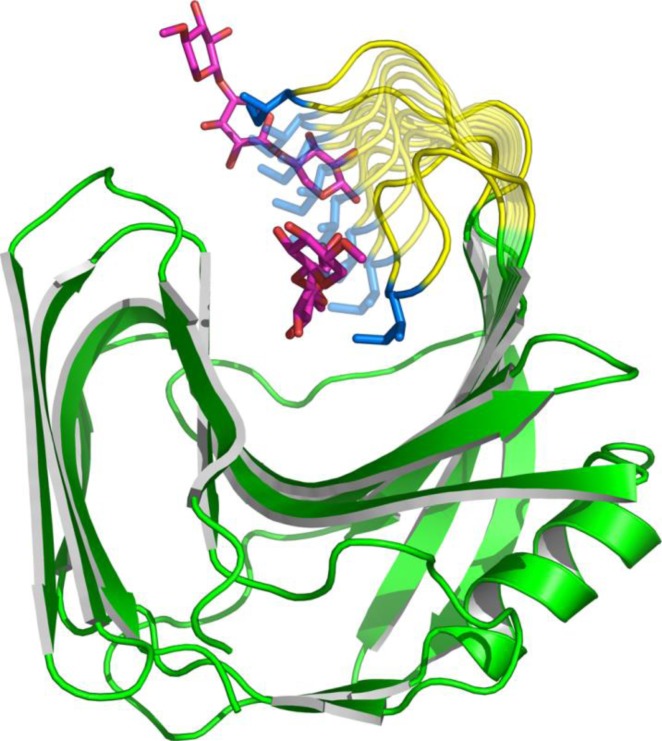
**Example of a trajectory of the xylotriose (pink) product released during the Tx-xyl thumb-loop motion (yellow); Ile116 is in blue.** For the thumb-loop, the first and last frames are in plain colors, intermediate frames are transparent. For clarity reasons, only the first and last frames of the xylotriose are displayed. The distance between the Cα of residue 116 in the first and last frames is 15.6Å.

To compute the coupled motions of the thumb-loop and the product, we used the 40 previously computed loop trajectories described above. For three of the four initial Ile116 rotamers, the xylotriose product was progressively pushed out of the cleft as the thumb-loop moved upwards (an observation that was verified for the 10 trajectories of each rotamer). On the contrary, the use of the fourth rotamer (also for the set of 10 trajectories) failed to reveal xylotriose release. Finer analysis of the results revealed that release of xylotriose was driven by two major effects. First, the modeled movement of the thumb-loop occurs in a rather narrow region of the catalytic cleft (approximately 6Å wide to be compared with the xylose moiety which is ∼4Å wide). During the movement, the tip of the thumb-loop further narrows the cleft and drastically reduces the empty volumes available for bound molecules. Secondly, regarding the position of Ile116, the loop motion relocates this residue from a position beneath the xylotriose molecule to a final position that is above that of the hydrolytic end product. Therefore, departure of the xylotriose product is a prerequisite for the upward movement of the thumb, which in the *in silico* simulation appears to flick the trisaccharide out of the active site. An example of this product release process is shown in Figure 7.

### Impact of in silico mutations at position 116 on xylotriose reaction product release

To investigate the role of residue 116 in the product release mechanism, Tx-xyl was submitted to *in silico* mutagenesis. Among the 19 possible mutations, five mutants were created: Tx-xyl-A, Tx-xyl-C, Tx-xyl-G, Tx-xyl-S and Tx-xyl-T, which correspond to mutants bearing Ala, Cys, Gly, Ser and Thr respectively at position 116. These side-chain alterations were chosen, not only because they represent variability in biochemical properties and spatial occupancy, but also because none of these side-chains naturally occur at position 116 in known GH11 xylanases. Additionally, very bulky side-chains were not considered in order to avoid steric clashes that might block thumb-loop mobility.

As before, the motions of the mutated thumb-loops were first computed and analyzed using apo-enzymes, before examining product expulsion in xylotriose-bound enzymes. For each mutant, rotamers that were found to be compatible with the upward motion of the thumb-loop were selected. Accordingly, one rotamer was selected for Ala and Gly, two for Cys and three each for Ser and Thr. Afterwards, trajectories of the different thumb-loops were sampled 10 times.

In agreement with the results obtained for the wild type thumb-loop motion, the mobility of the mutated thumb-loops was globally similar, consisting of the backward and upward motions. Once this was established, xylotriose release was simulated on the basis of the previously computed trajectories for each mutant. Moreover, assuming that the occurrence of all rotameric conformers is equally probable, the ability of the thumb-loop to perform xylotriose expulsion was estimated (expressed as the ratio between the number of observed ejections and the total number of trajectories) (Table 2). Accordingly, two groups of mutants were distinguished: Ala, Gly, Ser (≤10% probability of expulsion) and Ile, Cys, Thr (≥33% probability of expulsion).

**Table 2:** Comparison of *in silico* probability of xylotriose product release and *in vitro* relative activity depending on the residue at position 116. The last row displays *in vitro* relative activity, derived from the catalytic efficiency *k*_cat_ / *K*_M__(app)_ of each enzyme in comparison to the activity of the wild type enzyme taken as reference (100%) from Table 3.

Enzyme name	Residue at position 116	Side-chain structure (X stands for amino acid main chain)	Amino acid volume (Å^3^) [51]	*In silico* probability of xylotriose product releasing	*In vitro* relative activity (see Table 3)
Tx-xyl	Ile	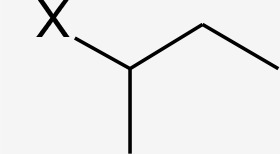	170	75%	100%
Tx-xyl-A	Ala	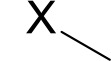	90	10%	12.3 ± 3.3%
Tx-xyl-C	Cys	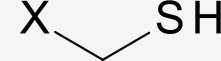	109	50%	108.3 ± 21.1%
Tx-xyl-G	Gly	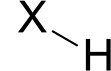	60	0%	0.4 ± 0.1%
Tx-xyl-S	Ser	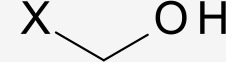	89	0%	1.4 ± 0.5%
Tx-xyl-T	Thr	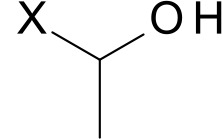	116	33%	12.7 ± 3.7%

A more detailed analysis of the results revealed that the upward motion of the thumb-loop gives rise to two possible outcomes, which correspond to high and low probabilities of product release in the *in silico* studies. In the first case, the thumb-loop reduces empty volumes to such an extent that xylotriose can no longer be accommodated in the cleft and the trisaccharide is released (through an apparent flicking action of the thumb-loop). Alternatively, the second scenario leaves sufficient space in the catalytic cleft, such that the thumb-loop only slightly contacts the surface of the xylotriose and fails to eject it. The latter scenario occurred most probably when smaller residues (Ser, Gly, Ala) were present at position 116, suggesting that steric interactions are a major determinant of the interaction between the thumb tip and the trisaccharide product. In particular for Gly116, the absence of side-chain allows the main chain to adopt more flexibility, leading to an even less bulky thumb-loop tip. Correlating the volume of the various side-chains used in the study with the probability of product release (derived from the simulation) (Table 2) indicates that a threshold volume might exist. However, because this correlation is not quantitative, other factors that might influence product release cannot be excluded (see Discussion).

### Impact of in vitro mutations at position 116 on catalytic activity

To further analyze the role of residue 116 in the activity of GH11 xylanases, *in vitro* site-directed mutagenesis was used to create the mutant enzymes Tx-xyl-A, Tx-xyl-C, Tx-xyl-G, Tx-xyl-S and Tx-xyl-T and then the catalytic parameters *k*_cat_ and *K*_M__(app)_ were measured (Table 3). Comparison of the values of the ratio *k*_cat_ / *K*_M_ shows that these differ by up to two orders of magnitude and closer examination reveals that the value of *k*_cat_ is most affected by mutation of residue 116. The mutants Tx-xyl-C, Tx-xyl-T and Tx-xyl-A were most active (Table 3), with Tx-xyl-C being slightly more active than the wild-type enzyme. The mutants Tx-xyl-G and Tx-xyl-S were least active (approximately 100-fold lower than the wild type enzyme). To facilitate comparisons, relative activities have been calculated, using wild type Tx-xyl as reference (100%) (Table 2). Overall, *in vitro* data show that the enzyme activity is strongly affected by a single mutation at position 116, and that a relationship exists between the experimentally measured activity and the probability of loop-induced product release measured *in silico*. None of the simulated loop motions for Tx-xyl-G and Tx-xyl-S led to product release, which is in good agreement with very low relative reactivity measured *in vitro*. Similarly, for Tx-xyl-A only 10% of the loop motions provoked product release, which is coherent with the weak *in vitro* activity of this mutant. Although qualitatively satisfying, our *in silico* approach overestimated product release for Tx-xyl-T: one third of loop trajectories yielded xylotriose release, whereas the measured activity is only slightly higher than that for Tx-xyl-A. In contrast, the computed probability of product release underestimated the relative activity for Tx-xyl-C compared to wild type Tx-xyl. Cys at position 116 ranks second (after the wild type residue Ile) in terms of probability of product release, while the activity of the mutated enzyme slightly improves that of the native enzyme. The discussion below provides further explanations to these results.

**Table 3:** Catalytic parameters of wild-type Tx-xyl and mutants.

Enzyme name	Residue at position 116	*k*_cat_ (s^-1^)	*K*_M__(app)_ (g×L^-1^)	*k*_cat_ / *K*_M__(app)_ (L×g^-1^×s^-1^)
Tx-xyl	Ile	8385 ± 109	1.8 ± 0.2	4684 ± 630
Tx-xyl-A	Ala	1869 ± 69	3.2 ± 0.3	575 ± 75
Tx-xyl-C	Cys	8769 ± 269	1.7 ± 0.1	5075 ± 304
Tx-xyl-G	Gly	116 ± 7	6.2 ± 0.7	18.5 ± 3.2
Tx-xyl-S	Ser	503 ± 42	7.9 ± 1.2	64 ± 15
Tx-xyl-T	Thr	4310 ± 245	7.2 ± 0.7	596 ± 95

## Discussion

### Conservation of the tip of the thumb-loop in GH11 xylanases

A comprehensive PFAM [52] multiple alignment (516 sequences, accession number PF00457, consulted in late 2011) of GH11sequences revealed that residue 116, or its equivalent, is highly conserved in the GH11 family. Isoleucine is the most frequent occurrence at this position, although it is occasionally replaced by other hydrophobic residues: valine or leucine. Moreover, examination of available 3D structures of GH11 xylanases reveals that Ile116 (or its counterparts) is spatially conserved at the tip of the thumb-loop [53]. Nevertheless, our work has shown that non natural residues (e.g. cysteine) can replace isoleucine at position 116, while conserving a similar level of catalytic efficiency. Substitution by Ala or Thr also yielded functional enzymes, albeit with reduced activity. However, not all amino acid side-chains can functionally replace Ile. In particular, Gly and Ser, whose side-chains are not radically different from those of Ala and Thr respectively, are unsuitable replacements, because they yield catalytically-crippled enzymes. Overall, our results demonstrate that residue 116 (or its equivalent) critically influences catalysis in GH11 xylanases, with certain mutations at this position leading to > 100-fold loss in activity. In other words, the importance of the amino acid side-chain at position 116 is comparable to that of the catalytic residues [38] or of the residue occupying position 7 (or its equivalent) [35], whose mutation severely reduces activity.

### Factors affecting the reaction product release by the thumb-loop

In this study, the *in silico* analysis of several mutants has revealed a correlation between the volume of side-chain residue present at position 116 and the ability of the thumb-loop to assist product release (Table 2). When the side-chain volume is below a threshold value of approximately 100 Å^3^, *in silico* product release appears to be compromised. However, the correlation is not quantitative, and in addition to steric considerations, other physicochemical factors might affect the simulated release probabilities for the different mutants. For example, Thr and Ser are polar residues bearing hydroxyl moieties (Table 2), which could act as donors, forming hydrogen bonds with Arg71 (Nɛ2) and/or Trp69 (Nɛ1). Presumably, such an interaction would temporarily lock the thumb-loop position, thus slowing its motional frequency. On the other hand, the greater hydrophobicity of Cys, Ile and Ala implies that thumb-loop tips bearing any of these amino acids would be less solvent-exposed than those with a Ser or Thr at position 116. Moreover, hydrophobic side-chains at position 116 would better repulse xylosyl moieties, thus alternatively providing the driving force for product expulsion and the tendency of the thumb-loop's tip to return to the bottom of the catalytic cleft after product releasing. Additionally, the highly conserved Trp7 facing residue 116, on the opposite side of the catalytic cleft, could favor the upward and backward motions through the creation of hydrophobic interactions.

### Importance of the thumb-loop for Tx-xyl catalysis

**Figure 8:**
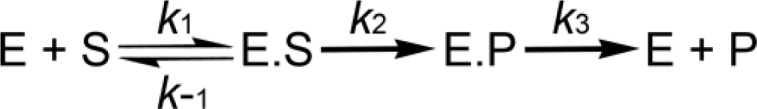
Catalytic reaction scheme of Tx-xyl with a putative kinetically-controlled product releasing.

Taken together, our results suggest that the thumb-loop plays an active role in catalysis. According to the classical scheme that is used to describe the double-displacement mechanism of retaining glycoside hydrolases (Figure 8), the first two steps of the reaction, which lead to enzyme glycosylation, are governed by the ratio *k*_cat_ / *K*_M_, which contains the constants *k*_1_, *k*_-1_ and *k*_2_ (Eq.1), while *k*_cat_ contains the glycosylation and deglycosylation constants, *k*_2_ and *k*_3_ respectively (Eq. 2). When the deglycosylation step is rate-limiting, it is expected that both *k*_cat_ (Eq. 2) and *K*_M_ (Eq. 3) will be lowered, in the latter case because of accumulation of the glycosyl-enzyme intermediate.

$$k_{cat}/K_{M}=\frac{k_{1}\cdot k_{2}}{k_{2}+k_{-1}}$$

$$k_{\text{cat}}=\frac{k_{2}\cdot k_{3}}{k_{2}+k_{3}}$$

$$K_{M}=\frac{k_{3}\cdot \left(k_{2}+k_{-1}\right)}{k_{1}\cdot \left(k_{3}+k_{2}\right)}$$

Considering the Tx-xyl variants, the *in vitro* mutagenesis data (Table 3) reveal that mutations at position 116 mainly influence *k*_cat_ (up to 70-fold decrease), while *K*_M__(app)_ is moderately changed (no more than a 5-fold variation). Therefore, these results show that the mutations mainly influence *k*_2_ and/or *k*_3_ (Eq. 2), but do not affect the initial association of the enzyme and its substrate. It is reasonable to postulate that *in vitro* mutations of residue 116 do not directly interfere with the catalytic functions of residues Glu76 and Glu169 (*k*_2_), which are unlikely to be affected by changes at the tip of the thumb-loop. Moreover, it is probable that these mutations affect, at least in a minor way, sugar binding in the distal (– 3) and (– 2) subsites and have a strong impact on product release (*k*_3_).

Accordingly, comparison of the values of *k*_cat_ of the mutant Tx-xyl-G (crippled enzyme) and of the wild type enzyme Tx-xyl allows us to estimate that the presence of a functionally pertinent amino acid side-chain at position 116 accelerates the reaction 70-fold. Obviously, this interpretation will be valid only if the structure of the mutated thumb-loop remains unaltered. Although no structural confirmation is provided here, we believe that this assumption is legitimate, because the fold of the thumb-loop is determined by a complex, strong internal hydrogen bond network that does not involve residue 116 [41].

Overall, the *in vitro* study performed extends our understanding of the functional role of the thumb-like loop in GH11 xylanases, revealing that in addition to its important role in substrate selectivity [41, 54], it is also important for glycosylation/deglycosylation and product release.

### A hypothetical model for the catalytic cycle in Tx-xyl

As discussed above, the *in silico* and *in vitro* results presented herein indicate that product release in Tx-xyl, and more generally in GH11 xylanases, is at least partially governed by steric encumbrance, together with the electrostatic interactions that link the glycone product to the (– 1) and (– 2) subsite determinants. Taking the results from this work together with previous data, we propose a putative comprehensive catalytic reaction mechanism. In the initial stage, it is expected that the thumb-loop will adopt an open configuration, rather like the one observed recently [42]. While in this configuration, the thumb-loop contacts the xylan or xylooligosaccharide substrate and begins to close down towards the catalytic cleft, binding interactions optimally positioning the substrate for catalysis. As previously suggested, in this closed position, the thumb-loop fills in the catalytic cleft, making it too narrow to accommodate other substrates such as gluco-oligosaccharides [41]. Hydrolysis, mediated by the catalytic dyad Glu76 / Glu169, results in breakage of the glycosidic bond linking the xylosyl moieties bound in (– 1) and (+ 1) subsites and the formation of two products. The aglycone product (bearing the nascent non-reducing end) leaves the catalytic cleft after the glycosylation step, whereas departure of the glycone product (bearing the nascent reducing end) occurs after deglycosylation step. At this stage, the lowered binding energy of the glycone product bound in subsites (– 1) to (– 3) (Fig. 3) allows the thumb-loop to move upwards, either in a passive manner or by transferring energy to the reaction product, which is sandwiched between the extremity of the thumb-loop and Trp7 in the (− 2) subsite. In the latter case, the upward movement of the thumb-loop might be likened to that of a lever, forcing product expulsion as the loop regains its initial open configuration, and thus returning the enzyme to a ground state. In the case of mutations at position 116 that affect *k*_cat_, it can be hypothesized that the leverage function is impaired, and thus glycone product departure occurs less efficiently. Obviously, while this model accounts for the critical role played by residue 116 and provides a satisfactory explanation for the importance of thumb-loop mobility, it does not offer any explanations of the energetic forces that might be involved.

### The pertinence of a combined robotics-molecular modeling strategy

It has been previously established that robotics path-planning algorithms are efficient conformational filters for investigating protein loops motions [11–13]. Indeed, conformations that satisfy a set of geometric constraints, including loop closure, correct bond geometry (for covalent and hydrogen bonds) and absence of steric clashes, are a reasonably good approximation of energetically feasible conformations, and thus motions computed with robotics-inspired algorithms can be considered as an approximate representation of possible conformational transitions.

Here, for the first time, robotics techniques, molecular modeling tools and mutational studies have been combined to investigate the movement of a prominent loop that is characteristic of GH11 xylanases. The computational processes were very fast, with the geometric path-planning step requiring just a few seconds to deliver a Tx-xyl thumb-loop trajectory (on a single processor). Comparatively, the molecular modeling step, involving energy minimization, took approximately 30 minutes for each trajectory analyzed. Overall, several trajectories of different thumb-loop mutants were computed in just a few hours, which is very rapid in comparison to other simulation or experimental methods. These calculation times clearly demonstrate that the described protocol could be used as a predictive tool to provide preliminary dynamical explanations of enzyme behavior. We must keep in mind that the essential strength of this protocol is to quickly provide molecular hypotheses that should be refined by more accurate MD simulations with *in silico* mutations. Ultimately and much more interestingly, mutations are suggested for experimental validations.

In the present case, the confrontation of the *in silico* results with experimental data demonstrated the reliability of the method, although drastic hypotheses were imposed to speed up the calculations and could possibly bias the global interpretation. The major imperfections of our process are following:

- the robotics-based conformational exploration was only applied to the loop, not to the whole protein;
- the statistics are based only on ten paths for each conformer;
- the selected conformers for each mutation at 116 position are assumed to be equiprobable, which is not necessarily true for residues having a long side-chain in particular;
- the energy minimizations are essentially applied to the substrate, in order to fit the room available along the loop trajectory.

Despite the shortcomings of our method, the different thumb-loop conformations have been found to have equal probabilities of occurrence, which is in accordance with the MD simulations previously performed on other GH11 xylanases [43, 44]. More remarkably, we have observed a very satisfying qualitative correlation between the *in silico* estimated probability of xylotriose releasing and *in vitro* measured relative activity (Table 2). But probably, the major success of this protocol was to suggest Ile116Cys mutation which naturally does not exist in GH11 xylanases sequences. As far as we know, this protocol is the only predictive tool capable to provide a dynamical interpretation of enzyme behaviour. Until now, our investigations have only focused on residue 116, but the same method could be applied to more extensive mutational studies (*e.g*. multiple mutations, deletions, or extensions) of the thumb-loop and could be transferred to other enzymes involving a loop motion during catalysis. Furthermore, we expect that the full structural characterization of the mutants described here will provide a complete validation of this approach.

## Abbreviations

GH: glycoside hydrolase

Xyl-11: glycoside hydrolase family 11 xylanase

Tx-xyl: glycoside hydrolase family 11 xylanase from *Thermobacillus xylanilyticus*
